# Embraced by eIF3: structural and functional insights into the roles of eIF3 across the translation cycle

**DOI:** 10.1093/nar/gkx805

**Published:** 2017-09-14

**Authors:** Leoš Shivaya Valášek, Jakub Zeman, Susan Wagner, Petra Beznosková, Zuzana Pavlíková, Mahabub Pasha Mohammad, Vladislava Hronová, Anna Herrmannová, Yaser Hashem, Stanislava Gunišová

**Affiliations:** 1 Laboratory of Regulation of Gene Expression, Institute of Microbiology ASCR, Videnska 1083, Prague 142 20, the Czech Republic; 2 CNRS, Architecture et Réactivité de l’ARN UPR9002, Université de Strasbourg, 67084 Strasbourg, France

## Abstract

Protein synthesis is mediated *via* numerous molecules including the ribosome, mRNA, tRNAs, as well as translation initiation, elongation and release factors. Some of these factors play several roles throughout the entire process to ensure proper assembly of the preinitiation complex on the right mRNA, accurate selection of the initiation codon, errorless production of the encoded polypeptide and its proper termination. Perhaps, the most intriguing of these multitasking factors is the eukaryotic initiation factor eIF3. Recent evidence strongly suggests that this factor, which coordinates the progress of most of the initiation steps, does not come off the initiation complex upon subunit joining, but instead it remains bound to 80S ribosomes and gradually falls off during the first few elongation cycles to: (1) promote resumption of scanning on the same mRNA molecule for reinitiation downstream—in case of translation of upstream ORFs short enough to preserve eIF3 bound; or (2) come back during termination on long ORFs to fine tune its fidelity or, if signaled, promote programmed stop codon readthrough. Here, we unite recent structural views of the eIF3–40S complex and discus all known eIF3 roles to provide a broad picture of the eIF3’s impact on translational control in eukaryotic cells.

## OVERVIEW OF THE TRANSLATIONAL CYCLE

**Figure 1.**
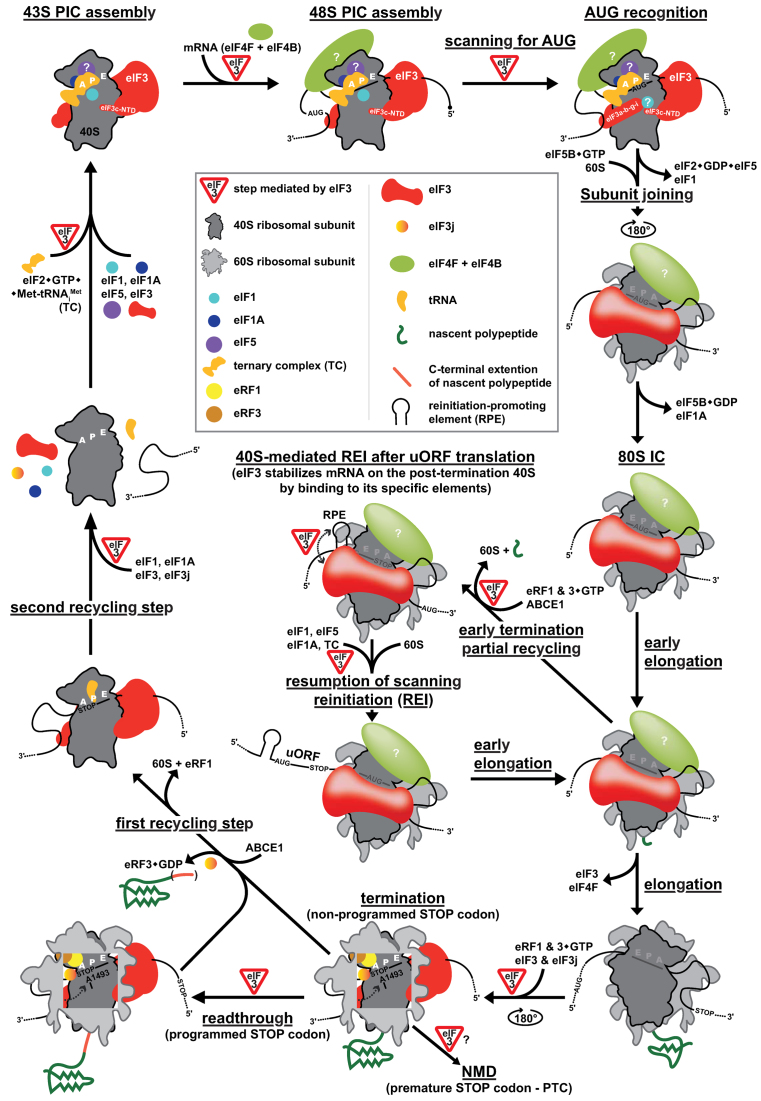
Schematics of the entire translational cycle with ‘detours’ for: (1) reinitiation, (2) programmed stop codon readthrough and (3) the Nonsense-mediated decay pathway, highlighting the role of eIF3 at the individual steps. For details, see the main text.

To begin a translational cycle, mRNA has to be brought to the ribosome in a way so that the start of the coding sequence that it carries is properly identified (reviewed in (1,2)). This is ensured by the initiator methionyl tRNA (Met-tRNA_i_^Met^) whose CAU anticodon is complementary to the most common initiation codon - AUG. Met-tRNA_i_^Met^ is delivered to the ribosome as a part of the so-called ternary complex (TC) together with the translation initiation factor 2 (eIF2) bound to a GTP molecule. Binding of the TC is aided by several other eIFs such as eIF1, 1A, 3 and 5 (Figure 1). Completion of this step results in a formation of the so-called 43S pre-initiation complex (PIC). Another co-operative role of eIFs 1, 1A, 3 and 5 is to prepare the small ribosomal subunit for mRNA docking by opening the 40S mRNA binding channel, initially believed to be mediated only by eIF1 and 1A. mRNA comes pre-bound by the group of the eIF4F factors, out of which eIF4E contacts the mRNA’s 5′ 7-methyl guanosine cap, as well as the scaffold protein eIF4G (Figure 1). eIF4G further interacts with the helicase eIF4A and poly(A)-binding protein PABP1, and together with eIF3 represents the major driving force in mRNA recruitment and accommodation in the 40S mRNA binding channel. Binding of the 43S PIC to mRNA close to its cap structure yields the 48S PIC, which subsequently begins inspecting the sequence of nucleotides downstream of the cap in the process known as scanning. Scanning requires the action of helicases such as eIF4A (working together with its stimulatory factors eIF4B and eIF4H) and DHX29 (occurring only in higher eukaryotes) to unwind mRNA secondary structures for the ribosome to move smoothly along the 5′ UTR till the start codon (usually the first AUG) has been recognized. The AUG recognition triggers a series of intricate events and conformational changes in the PIC involving irreversible GTP hydrolysis on eIF2 co-operatively mediated by eIF5, eIF1, eIF1A and eIF3. This results in the closure of the 40S mRNA binding channel and ejection of most of the initiation factors from the 48S PIC (for example of the eIF2•GDP•eIF5 assembly) (1) (Figure 1). In contrast, accompanying these changes, the eIF1A binding to the 48S PIC becomes tighter. eIF5B bound to GTP then mediates subunit joining at the expense of the second and last GTP hydrolysis in the entire initiation phase. Ejection of eIF5B hand in hand with eIF1A marks the end of the initiation phase leaving the 80S initiation complex behind poised for elongation (3) (Figure 1). For the next round of initiation, eIF2B (the guanine nucleotide exchange factor - GEF) must first out-compete eIF5 from the eIF2•GDP•eIF5 assembly in order to mediate the exchange of GDP for GTP to bring eIF2 back to its active (GTP-bound) state (4,5).

The elongation phase consists of a string of repetitive events mediated by elongation factors eEF1A (a GTPase mediating the recruitment of aminoacyl-tRNAs to the A-site of the elongating ribosome), eEF1B (GEF for eEF1A) and eEF2 (a GTPase promoting translocation of the 80S ribosome by one triplet at a time), the purpose of which is to add one amino acid residue *per* each triplet following AUG into the growing polypeptide chain (Figure 1) (reviewed in (6)).

The termination phase commences with the stop codon slippage into the A-site during the last round of the eEF2•GTP-mediated translocation. In eukaryotes, all three existing stop codons are recognized by a single release factor eRF1 that comes in a complex with the GTPase eRF3 (reviewed in (7)). According to the most recent model, eRF3 senses the proper accommodation of eRF1 at the A-site occupied by the stop codon (8) (Figure 1). This triggers GTP hydrolysis on eRF3, which then leaves the pre-termination complex to make room for the recycling factor called ABCE1 (RLI1 in budding yeast). Binding of ABCE1, a member of the ATP-binding cassette (ABC) family of proteins (actually, it can bind and hydrolyze any NTP), promotes polypeptide release by pushing the GGQ motif of eRF1 into the peptidyl transferase center to kick off the hydrolysis of ester bond between the nascent polypeptide chain and the CCA end of the peptidyl-tRNA sitting at the P site. This NTP-independent step captures the production of a particular protein.

In order to complete the entire translational cycle and be able to start all over again, ABCE1 (RLI1 in yeast) hydrolyses its NTP and transforms the released energy into the mechanochemical force splitting the 80S termination complex into its individual subunits: 60S and 40S, the latter of which still contains de-acetylated P-site tRNA and mRNA (9–11) (Figure 1). Generally speaking, two scenarios are possible at this stage. (i) In case of long ORFs, the post-termination 40S-tRNA-mRNA complex is fully recycled by a joint action of canonical initiation factors eIF1, eIF1A and eIF3 or by eIF2D (known also as Ligatin or TMA64 in yeast), or by the heterodimer MCT1–DENR (known as TMA20 and TMA22 in yeast) corresponding to N-terminal and C-terminal regions of eIF2D, respectively (12,13). (ii) If the translated ORF is very short (up to 10 codons in yeast and 30 codons in higher eukaryotes) and surrounded by specific *cis-*acting features, the post-termination small ribosomal subunit may remain bound to the mRNA with help of eIF3 (and perhaps also of eIF4G), resume scanning and reinitiate protein synthesis from an AUG downstream of the stop codon (Figure 1) (reviewed in (2,7,14)).

In some specific cases, the stop codon does not signal the actual end of protein synthesis. This phenomenon is called stop codon readthrough or nonsense suppression and occurs when a near-cognate aminoacyl-tRNA (nc-tRNA) or a natural suppressor tRNA (fully cognate with a given stop codon) wins the otherwise uneven competition with eRF1 over the corresponding stop codon (Figure 1) (reviewed in (15,16)). It can be ‘spontaneous’ and thus relatively infrequent or it can be programmed to C-terminally extend the protein of interest as a response to specific environmental changes demanding an alteration of the corresponding protein's properties (17). Stop codon readthrough can also occur at a premature termination codon (PTC) within the coding region of a given gene, which is actually desirable because it can prevent the action of nonsense-mediated decay (NMD) pathway by ensuring the synthesis of a full-length protein (18). Numerous factors influence the efficiency of readthrough, such as for example the identity and the nucleotide context of the stop codon, the identity of the last two amino acids incorporated into the polypeptide chain, the identity of the P-site tRNA, the presence of stimulatory elements downstream from the stop codon (15), and last but not least, the identity and concentration of nc-tRNAs (19) and, rather unexpectedly, the presence of eIF3 in the pre-termination complex (20,21).

Here, we provide novel insights into all just described phases of translational cycle, as well as its modifications, from the perspective of the multitasking eukaryotic initiation factor eIF3.

## THE ROLE OF eIF3 IN GENERAL TRANSLATION INITIATION

**Figure 2.**
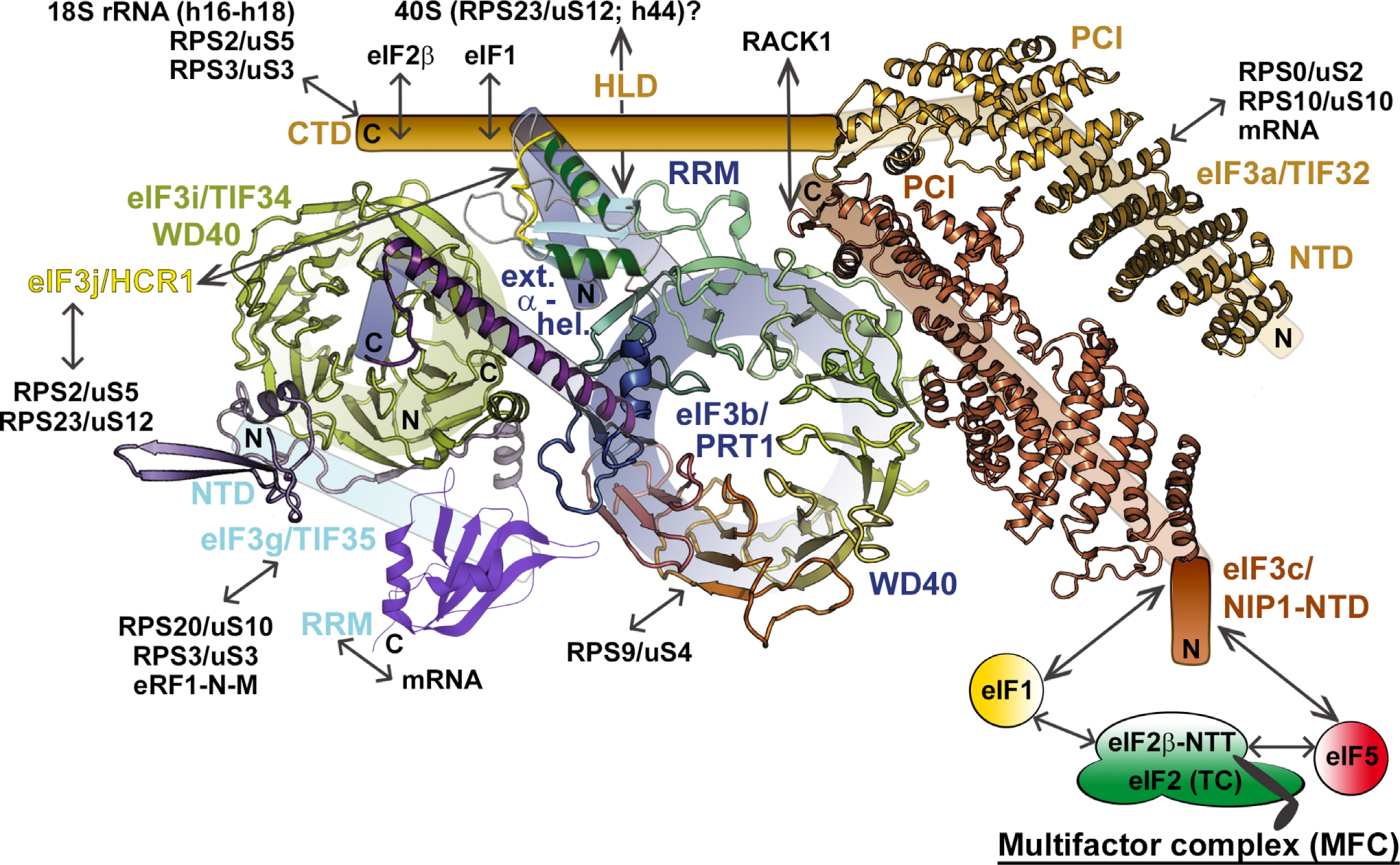
Schematics of the *S. cerevisiae* eIF3 complex. A 3D model of yeast eIF3 and its associated eIFs in the Multifactor complex (MFC) composed together to the best possible fit from all available structures of individual domains; namely the X-ray structure of the yeast i/TIF34 (full length)—g/TIF35-NTD (*S.c*. residues 1–135)—b/PRT1-extended-α-helix (*S.c*. residues 655–698) complex, the X-ray structure of the β-propeller (formed by nine WD40 repeats) of the middle domain of b/PRT1 (residues 132–626), the X-ray structure of the mutually interacting PCI domains of a/TIF32 (residues 1–496) and c/NIP1 (residues 251–812) (all taken from (64)); the NMR structure of the interaction between the RRM of human eIF3b (*H.s. residues* 170–274) and the N-terminal peptide of human eIF3j (*H.s. residues* 35–69) (37), and the NMR structure of the C-terminal RRM of human eIF3g (*H.s*. residues 231–320) (38). Arrows indicate all known interactions of eIF3 domains with other eIFs, ribosomal proteins and mRNA (see text for further details). NTD, N-terminal domain; CTD, C-terminal domain; HLD, HCR1-like domain; RRM, RNA recognition motif; PCI, PCI domain; WD40, WD40 domain; TC, ternary complex (composed of eIF2•GTP•Met-tRNA_i_^Met^).

**Figure 3.**
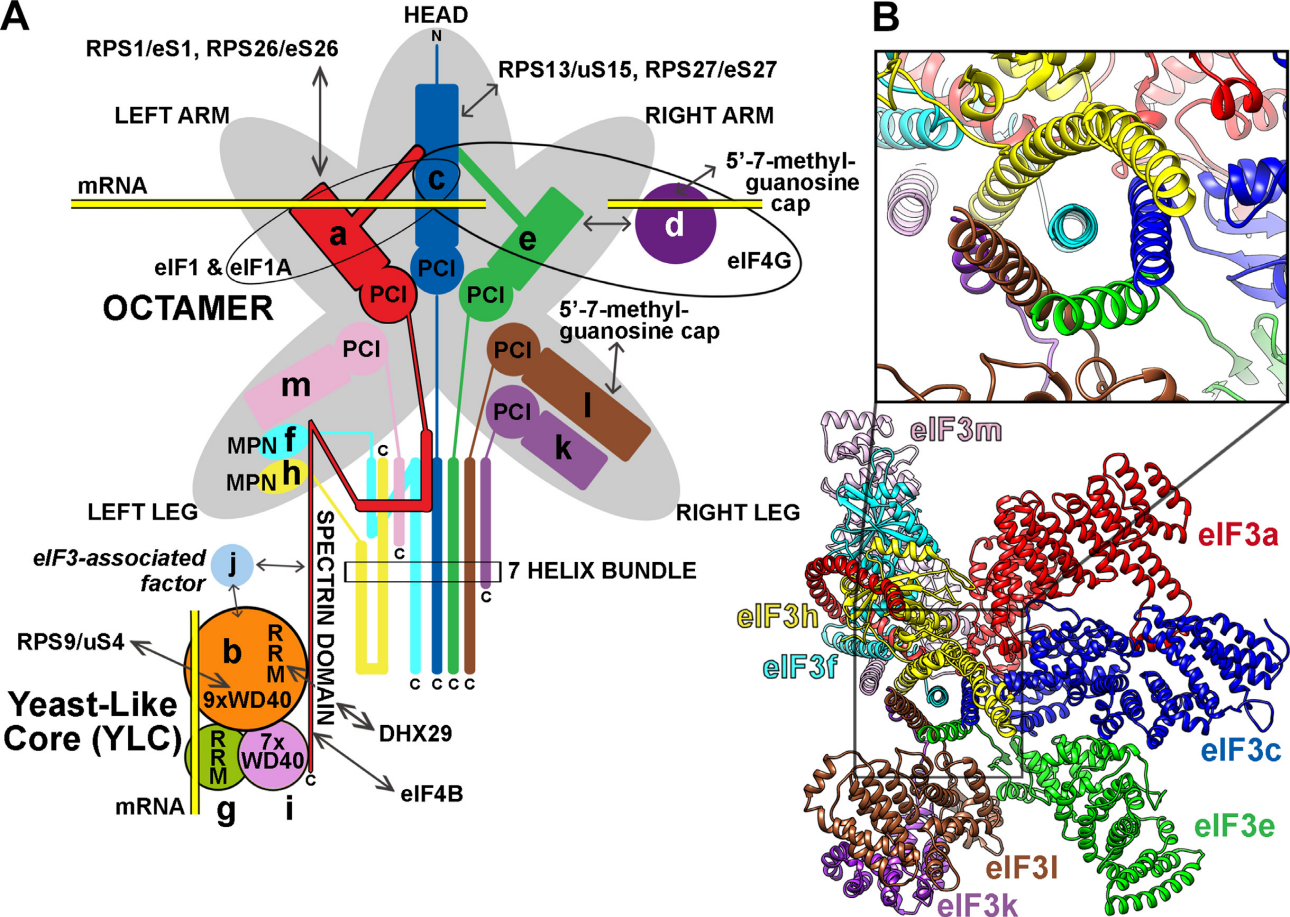
Schematics of the human eIF3 complex. (**A**) A schematic model of human eIF3 was adapted from (25). The eIF3 subunits forming the PCI/MPN octamer with the anthropomorphic shape are indicated by the grey background. The rectangle marks the seven α-helices involved in formation of the 7-helix bundle shown in panel C. The Yeast-Like Core (YLC) comprising the eIF3 subunits a, b, g and i (defined previously by (41)) is depicted and so is the eIF3-associated factor eIF3j with arrows indicating its contacts with other eIF3 subunits. The upper right-hand side arrow indicates the interaction between eIF3e and eIF3d that attaches eIF3d to the rest of eIF3 (48,59,159). Arrows indicate all known interactions of eIF3 domains with other eIFs, ribosomal proteins and mRNA (see text for further details). (**B**) Polyalanine-level model of the eIF3 octamer core with the close-up view of the 7*-*helix bundle formed by subunits h, c, e, f, l and k (adapted from (59)).

Translation initiation factor 3 (eIF3) has been considered the largest and the most complex of all eIFs ever since its first isolation (for review see (22)), yet its complete structure has not been determined yet. The *Saccharomyces cerevisiae* eIF3 (*S.c*.-eIF3) comprises five core essential subunits (a/TIF32, b/PRT1, c/NIP1, i/TIF34, and g/TIF35) (Figure 2) that have corresponding orthologs in the more complex mammalian eIF3 (m-eIF3), which contains seven additional subunits (eIF3d, e, f, h, k, l and m) reaching the total of 12 (reviewed in (2,23)) (see Table 1). Out of those 12, 8 subunits form the so-called octamer (a, c, e, f, h, k, l, m); eIF3b, g and i assemble into the so-called Yeast-Like-Core (YLC) together with the C-terminal region of the otherwise octameric eIF3a; and the remaining peripheral eIF3d subunit attaches to eIF3 *via* eIF3e (24,25) (Figure 3A). Originally, the yeast j/HCR1 (26) and its mammalian ortholog eIF3j (27) were believed to represent the 6th and 13th subunit of eIF3, respectively; however, recent evidence strongly indicates that they rather represent eIF3-associated factors (having mostly eIF3-independent roles) than the *bona fide* eIF3 subunits. For example, yeast j/HCR1 was proposed to play more important role in termination than in initiation (20), mammalian eIF3j and mRNA were found to bind anti-cooperatively to the 43S PIC (28), whereas the eIF3 complex is one of key factors promoting mRNA recruitment to the PICs, etc.

**Table 1.** Overview of eIF3 subunits and of the eIF3-associated factor eIF3j across species

Subunit	Domains	*S. cerevisiae*			*S. pombe*			*N. crassa*			*A. thaliana*			*H. sapiens*		
		Named	M.W. (kDa)	Essential^a^	Named	M.W. (kDa)	Essential^b^	Named	M.W. (kDa)	Essential^b^	Named	M.W. (kDa)	Essential	named	M.W. (kDa)	Essential^a^
**eIF3a**	PCI, Spectrin HLD (yeast)	**TIF32**	110.3	E	**p107**	107.1	E	**p110**	120.2	E	**p114**	114.3	?	**p170**	166.6	E
**eIF3b**	WD40, RRM	**PRT1**	88.1	E	**p84**	84.0	E	**p90**	85.6	E	**p82**	81.9	?	**p116**	92.5	E
**eIF3c**	PCI	**NIP1**	93.2	E	**p104**	104.4	E	**p93**	98.4	E	**p110**	103.0/91.7	?	**p110**	105.3	E
**eIF3d**	Cap-binding pocket?	-	-	-	**MOE1**	62.6	N	**eIF3d**	65.0	E	**p66**	66.7	?	**p66**	64.0	E
**eIF3e**	PCI	-	-	-	**INT6**	57.1	N	**INT6**	51.1	N	**p48**	51.8	E*	**p48**	52.2	E
**eIF3f**	MPN	-	-	-	**CSN6**	33.3	E	**eIF3f**	39.7	E	**p32**	31.9	E**	**p47**	37.6	E
**eIF3g**	RRM, Zn finger	**TIF35**	30.5	E	**TIF35**	31.5	E	**p33**	32.4	E	**eIF3g**	32,7/35,7	?	**p44**	35.6	E
**eIF3h**	MPN	-	-	-	**p40**	39.8	N	**eIF3h**	40.4	N	**p38**	38.4	E***	**p40**	39.9	N
**eIF3i**	WD40	**TIF34**	38.7	E	**SUM1**	36.8	E	**TIF34**	38.8	E	**p36**	36.4	?	**p36**	36.5	E
**eIF3k**	PCI	-	-	-	-	-	-	**p25**	26.8	N	**p25**	25.7	?	**p28**	25.1	N
**eIF3l**	PCI	-	-	-	-	-	-	**eIF3l**	54.5	N	**eIF3l**	60.2	?	**p67**	66.7	N
**eIF3m**	PCI	-	-	-	**CSN7B**	45.1	E	**eIF3m**	49.7	E.	**eIF3m**	46.8	?	**GA17**	42.5	E
**associated factor**	-	**HCR1**	29.6	N	**p35**	30.5	N	**HCR1**	30.3	N	**eIF3j**	25.5	?	**p35**	29.1	N
**eIF3j**																

^a^Essential: DEG, a Database of Essential Genes (161).

^b^Essential: (162).

*Gametogenesis.

**Pollen germination and embryogenesis.

***Significantly reduced fertility.

Over several decades of intensive research (mainly in budding yeast), eIF3 has been demonstrated to promote nearly every step of translation initiation. Briefly, the current knowledge is that eIF3 keeps the 40S and 60S subunits apart (29), several domains of its subunits directly stimulate the TC and mRNA recruitment to the PICs (30–36) and subsequently control the rate and processivity of scanning, as well as the fidelity of the start codon selection (30,31,35,37–44). eIF3 is apparently not actively involved in the subunit joining step even though it persists bound to the 80S initiation complex during early elongation (45–47). The breadth of the eIF3 roles in translation initiation most likely emanates from its complexity, as well as from the fact that it directly interacts with several other eIFs and to some degree co-ordinates their placement and functional conformations on the surface of the small ribosomal subunit (reviewed in (1,2)).

## THE eIF3 STRUCTURE, ASSEMBLY AND PLACEMENT IN THE 48S PRE-INITIATION COMPLEX

Based on the most recent analysis of human and *N. crassa* 12-subunit eIF3 (25,41,48), as well as the earlier analysis of the 5-subunit S.c.-eIF3 (32,49–55), it seems likely that the eIF3 nucleation core is formed by the eIF3a and eIF3b subunits in most, if not all, organisms. They interact with each other via the N-terminal RNA recognition motif (RRM) of eIF3b and the C-terminal spectrin domain (the HCR1-like domain in yeast) of m-eIF3a (32,49,55,56) (Figures 2 and 3A). The extreme C-terminal end of eIF3b recruits eIF3g and eIF3i and their mutual interaction further fortifies this eIF3b–g–i module, at least in yeast (54). In mammals, the eIF3a spectrin domain most probably contributes to or takes over this fortification role by stabilizing the eIF3b–eIF3i interaction (56) (Figures 2 and 3A).

In less complex S.c.-eIF3, the C-terminal domain of eIF3b together with the N-terminal PCI domain of eIF3a interacts with eIF3c *via* its C-terminal and PCI domains, respectively, to complete the assembly (32,34,52) (Figure 2). In more sophisticated eIF3 complexes, the eIF3a subunit nucleates the formation of the octamer (Figure 3A); i.e. a structural scaffold that is shared by the functionally unrelated 19S proteasome lid, as well as the COP9 signalosome (57). The PCI/MPN octamer always contains six subunits with a PCI domain (for Proteasome-COP9 signalosome-eIF3) and two subunits with an MPN domain (for Mpr1-Pad1 N-terminal) (Figure 3A). Formation of the eIF3a–eIF3b nucleation core seems to be a strict prerequisite for the human octamer assembly *in vivo* (25), even though the octamer can be formed in a test tube from recombinant proteins (58). In fact, it is possible that the formation of the eIF3a–b–g–i subcomplex (YLC) precedes the nucleation of the octamer because the YLC was shown to exist free in cytoplasm in human cells knocked down for the core octamer eIF3c, f and m subunits (25,41). According to the most recent data, the first step of the human octamer nucleation consists of the simultaneous addition of the eIF3c^PCI^, f^MPN^ and m^PCI^ (and perhaps also of eIF3h^MPN^) subunits to eIF3a^PCI^ from the 3a/3b nucleation core (25). This way the network of interactions among MPN and PCI domains nicknamed the β-sheet arc starts to build along with the additional multiple point of contacts called the α-helical bundle, to which all octameric subunits contribute by at least one α-helix (48,59) (Figure 3A and B). Subsequently, eIF3e^PCI^ together with its tightly binding partner represented by the non-octameric eIF3d subunit joins, followed by two tightly interacting octameric partners eIF3k^PCI^ and eIF3l^PCI^ to complete the assembly (25,48). It is noteworthy that the structural analysis of the recombinant human eIF3 out of the context of the ribosome resolved only the octameric subunits adopting a five-lobed structure with appendages reminiscent of a head, two arms, and legs (24,60); the structure of the rabbit octamer in the highest available resolution is shown in Figure 3B (59). Taking into account that neither eIF3d nor the YLC subunits could be determined suggests that all non-octameric subunits might be rather flexible.

**Figure 4.**
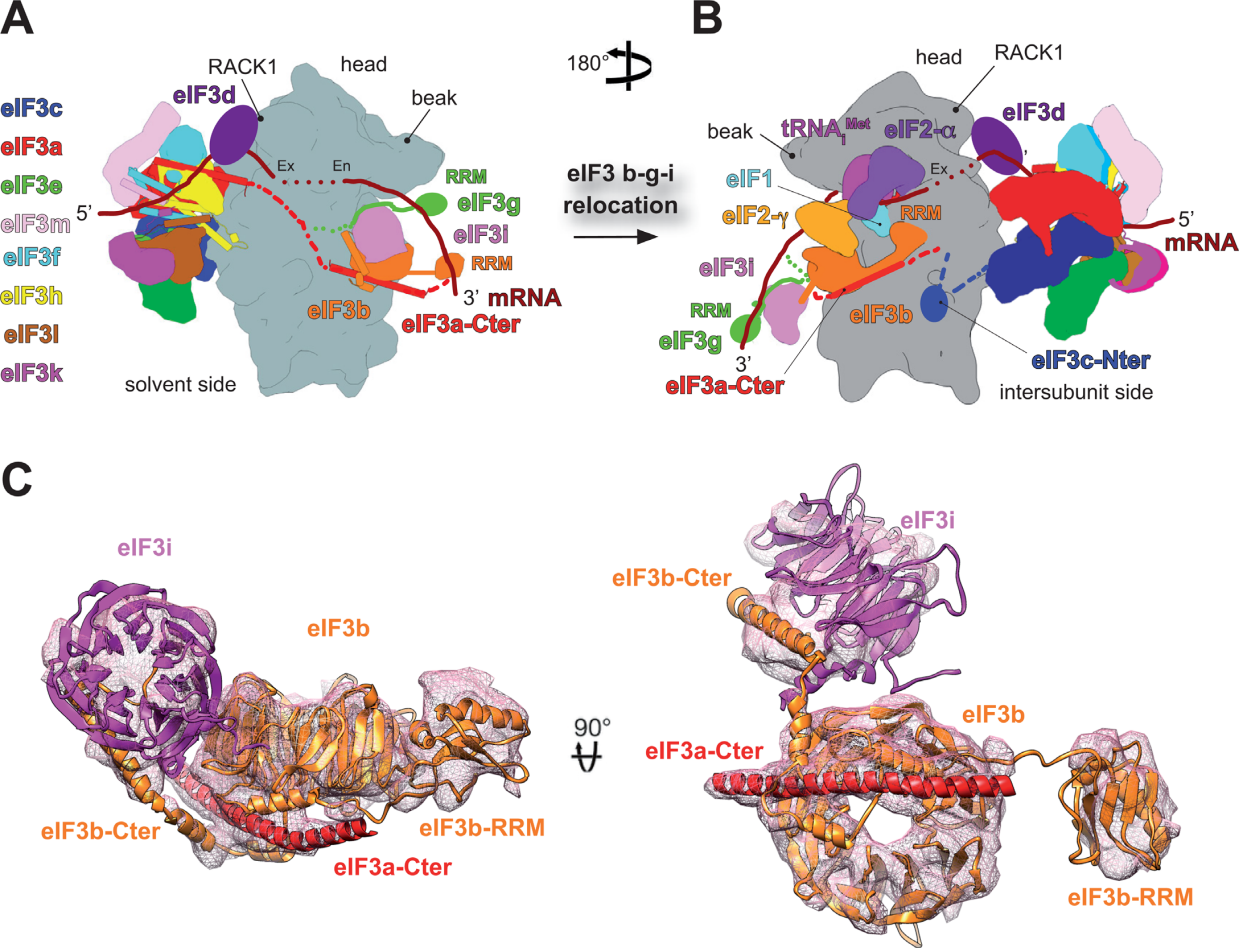
Schematic representation of the arrangement of eIF3 subunits in the two available conformations that are deduced from the available Cryo-EM analysis (adapted from (59)). (**A**) eIF3 binds to the solvent-exposed side with the octamer occupying the platform of the small ribosomal subunit connected with the eIF3b–g–i module (YLC)—sitting near the mRNA entry channel—*via* the extended C-terminal linker domain of eIF3a (a dashed red line indicates a predicted location of the eIF3a-CTD; placement of the eIF3g-RRM is also only our best guess) (59,63–65). Figure includes only those domains of eIF3 subunits for which the structures are known. The 40S subunit is depicted in grey surface; all other subunits are labelled and colored variably. The eIF3 helical bundles fortifying the intersubunit interactions are represented as cylinders. The predicted path of mRNA is shown in dark red; Ex and En—mRNA exit and entry channels, respectively. For details please see the main text. (**B**) In this conformation, the entire eIF3a-CTD–b–g–i module relocates from the solvent-exposed side to the intersubunit side, so that the eIF3b-RRM interacts with 18S rRNA and eIF1 and the eIF3b-propeller interacts with eIF2γ (66,70); a density presumably corresponding to the eIF3c-NTD residues 115–220 was in this structure identified not too far away from eIF1, where it could co-ordinate AUG recognition with other eIFs. Purely hypothetically, this could be the conformation that eIF3 adopts upon AUG recognition. Placement of the eIF3g–i unit held by the eIF3b-extended α-helix is only our best guess; for details please see the main text. (**C**) Atomic model of rabbit eIF3b (RRM, WD40 and the C-terminal extended α-helix domains - orange), yeast eIF3i-WD40 (purple) and a long α-helix (red) corresponding to a fragment of the C-terminal helical region of eIF3a. For details please see the main text.

The very first attempt to map the position of m-eIF3 on the small ribosomal subunit using the negatively stained EM images occurred in 1978 by the Freienstein's lab (61), followed by a similar study in the Frank's lab in 1992 (62). Both studies placed the eIF3 body close to the platform on the 40S solvent-exposed side. It took more than 20 years to visualize m-eIF3 in the 43S PIC in high-enough resolution to also predict the position of the YLC subunits (63) and later, in a more refined structure, to assign the observed densities to the individual subunits of m-eIF3 (59). Based on these structures, as well as on numerous biochemical reports that are in majority of cases consistent with the structural images, it is now clear that the major eIF3 body sits on the 40S solvent-exposed side, however, several of its subunits project into the ribosomal intersubunit side (Figure 4A and B). eIF3 thus embraces the small ribosomal subunit from both sides to control most, if not all, initiation reactions. In detail, the head and left arm represented by eIF3c and eIF3a were predicted to contact ribosomal proteins RPS13/uS15 and RPS27/eS27, and RPS1/eS1 and RPS26/eS26, respectively, occurring near the mRNA exit channel. Subunits eIF3e, h, k and l seem to stick out from the 40S-binding surface of the octamer into solution where they may mediate interactions with various partners to control the initiation rates in an mRNA-specific manner (see below).

Reconstruction and intensive integrative modeling of the yeast 40S–eIF1–eIF1A–eIF3 complex stabilized by crosslinking suggested that the eIF3a–eIF3c PCI heterodimer also sits near the mRNA exit channel, with the C-terminal portion of the eIF3a-PCI domain occupying the position of mammalian eIF3f and 3h subunits (64,65); the same arrangement was later observed even without cross-linking (66). In support, earlier biochemical experiments revealed that the N-terminal domain (NTD) of yeast eIF3a interacts with another ‘mRNA exit channel’ protein RPS0/uS2 (50,67) and contacts specific mRNA elements that promote translation reinitiation and are expected to reside in the vicinity of the mRNA exit channel (45–47). Similarly, m-eIF3a was crosslinked to 5′ UTR residues −14/−17 (relative to AUG) of model mRNA in the reconstituted 48S PICs and predicted to form a functionally important extension of the mRNA exit channel (68). Finally, using model mRNAs lacking contacts with the 40S entry or exit channels, we have recently uncovered a critical role for the a/TIF32-NTD in stabilizing mRNA interactions at the exit channel (35).

The outburst of recent cryo-EM reconstructions of both yeast and mammalian eIF3 in complex with the 40S subunit suggests that eIF3 can adopt several conformations depending on the actual initiation status. In particular, the eIF3b–g–i module is rather mobile, most probably thanks to the C-terminal domain of eIF3a that interacts with this module and thus may operate as a controllable mechanical arm (Figures 1, 4A and B). The first two mammalian structures predicted the densities observed near the mRNA entry channel to represent the β-propeller of eIF3b (thanks to the crystal structure showing that this propeller is atypically formed by 9 WD40 repeats (69)) with the disconnected, low-resolution density provisionally assigned as the eIF3b-RRM projecting laterally away from the 40S body (59,63,64) (Figure 4A). When viewed from the solvent-exposed side, the β-propeller of eIF3b is placed horizontally—touching RPS9/uS4 with the edge of one of its blades, whereas the seven-bladed WD40 β-propeller of eIF3i, which was assigned to another entry channel density thanks to the structure solved in (40), resides on the opposite side of the eIF3b β-propeller than the eIF3b-RRM in the vertical orientation (Figure 4C) (59). Both propellers are directly connected *via* the C-terminal extended α-helical domain of eIF3b, as shown before (40). The only part of the C-terminal mechanical arm of eIF3a that was resolved runs underneath the eIF3b β-propeller but it highly likely extends further towards the eIF3b-RRM domain (Figure 4A), because the major contact point between eIF3b and the eIF3a-CTD in both yeast and mammals is mediated *via* the eIF3b-RRM (32,54,56,70). Undoubtedly, it also connects with the octamer across the solvent side of the 40S ribosome towards its platform (see below); visualization of these particular contacts requires additional work (Figure 4A; red dashed lines). There was no apparent density for eIF3g in either of the structures.

In agreement, a nearly identical arrangement of the binary eIF3b–i subcomplex, represented by the RRM and both β-propellers attached to the eIF3a-CTD, was also deduced from the analysis of the yeast 40S–eIF1–eIF1A–eIF3 complex (64,65). In this yeast structure the eIF3g-NTD was assigned sandwiched between the eIF3i β-propeller and the 40S body (Figure 4A), in accord with the earlier work identifying contacts between yeast g/TIF35 and RPS3/uS3 and RPS20/uS10, occurring at or near the mRNA entry channel (38). In addition, in this structure the density underneath the eIF3b β-propeller corresponding to the eIF3a-CTD extends N-terminally towards the eIF3a–3c PCI heterodimer clearly demonstrating that the eIF3a-CTD links both the PCI and eIF3b–g–i modules residing at or near the exit and entry channels, respectively, as suggested before (40). The overall placement of the yeast trimeric eIF3b–g–i module attached to the eIF3a-CTD is consistent with the previously identified contacts between the a/TIF32-CTD and RPS2/uS5 and RPS3/uS3 (39) and helices 16–18 of 18S rRNA (50), and between the eIF3b β-propeller and RPS9/uS4 (69). It also agrees with the protection of h16 nucleotides from chemical or enzymatic cleavage by m-eIF3 (68). In this conformation, the eIF3a-CTD–b–g–i module seems well positioned to interact with incoming mRNA by extending the mRNA entry channel and modulating the rate and processivity of scanning for AUG recognition *in vivo*, as experimentally evidenced (37–40,43,51) (Figure 4A). In accord, the recent biophysical work implicated both the entry and exit channel modules of yeast eIF3 in performing highly-specific roles during initiation, some of which are co-operative (like stabilizing the assembly of the PICs and mRNA recruitment), while some others are module-specific (35). For example, alterations to the a/TIF32-CTD and the eIF3b–g–i module significantly slowed mRNA recruitment and mutations within the eIF3b–g–i module destabilized eIF2•GTP•Met-tRNA_i_ binding to the PIC, whereas alterations to the a/TIF32-NTD destabilized mRNA interactions with the PIC at the exit channel (35).

The most recent yeast cryo-EM structures revealed the yeast 48S PICs during mRNA scanning (in the so-called py48S open conformation) and right at the start codon recognition (in the py48S closed conformation) (66) (Figure 4B). Apart from the eIF3a–eIF3c PCI dimer occurring at the 40S platform, a drum-like shape density at the 40S intersubunit face was observed near the RPS23/uS12, h44, and the eIF2γ subunit, and tentatively attributed to the eIF3i β-propeller with the extended α-helix of eIF3b and the eIF3g-NTD. Other isolated densities on the interface side were provisionally attributed to different parts of the yeast eIF3a-CTD and eIF3c-NTD (one of these, predicted to represent the extreme NTD of eIF3c-NTD, occurred next to eIF1 residing near the P-site, based on the known contact between these two molecules (31)). The authors suggested that this rather robust conformational rearrangement (mainly with respect to the eIF3g–i unit) may occur on mRNA binding. However, this assignment was later challenged because the 9-bladed β-propeller of eIF3b aligned much better to the drum-like density in the original cryo-EM map (70). Furthermore, the eIF3b-RRM motif closely matched the density in a direct contact with eIF1 (originally predicted to represent the c/NIP1-NTD (Figure 4B). The density linking the β-propeller and the RRM of eIF3b was intuitively predicted to be composed of the mostly unstructured eIF3b linker sequence loosely bundled with the extended α-helical region of the eIF3a-CTD (70) (Figure 4B); as mentioned above, the eIF3a-CTD and eIF3b-RRM directly interact (49) and the yeast eIF3a-CTD also interacts with eIF1 (32)). In fact, the projection of the eIF3a-CTD into the intersubunit space was - thanks to its contact with the eIF2β - already proposed earlier (32,50). Since these reassignments are definitely correct ((1) and José Llácer Guerri, personal communication), the positions of eIF3g and eIF3i in these particular yeast conformations remain unknown.

It is important to note that in Simonetti *et al.* we made an attempt to assign another intersubunit-side based density that we observed in our near-native conditions directly in cell extracts to the mammalian eIF3g–i unit (70); however, this density later turned out to be ABCE1 (71,72). Nonetheless, taken into account that the interaction between the eIF3b-extended α-helix and the eIF3g–i unit is highly conserved, it seems very likely that the entire eIF3a-CTD–b–g–i module relocates from the solvent-exposed side to the intersubunit side, so that the eIF3b-RRM interacts with 18S rRNA and eIF1 and the eIF3b-propeller interacts with eIF2γ (Figure 4B). Whether this remarkable rearrangement occurs on mRNA binding, or on the onset of scanning, or on the irreversible GTP hydrolysis upon AUG recognition remains to be determined. The proposed contacts with eIF2γ and eIF1 may slightly favor the latter idea; the eIF3b-RRM could stimulate the GTP hydrolysis on eIF2γ and help to kick eIF1 out of the P-site, which is one of the most critical steps underlying the proper AUG selection (reviewed in (1)).

The NTD of eIF3c was shown to mediate eIF3 interactions with eIF1 and 5 in yeast (Figure 2) (30,31,53,73) and with eIF1 in mammals (24,74), and also proposed to project into the intersubunit side to coordinate AUG recognition with the latter eIFs (50). Consistently, crosslinking mass spectrometry data predicted an interaction of the N-terminal segment of eIF3c with eIF1 bound to the 40S platform (64), and a density presumably corresponding to the eIF3c residues 115–220 was identified not too far away from eIF1 in both the py48S-closed/open complexes (66) (Figure 4B). Furthermore, several studies proposed intricate molecular interactions among the eIF3c-NTD and eIFs 1, 2 and 5 within the PIC that would enable rapid scanning-arrest at the start codon by removing eIF1 away from the ribosomal P-site (30,31,42). Since the eIF3c-NTD holds eIF1 in the so-called eIF3–1–2–5 multifactor complex (Figure 2) (73) and most likely promotes eIF1 delivery to the PICs (perhaps directly releasing it into the P-site), an intriguing idea is that upon AUG recognition, the NTD of eIF3c rebinds eIF1 and clears it away from the P-site to irreversibly stall the initiation machinery at the correct AUG (2,31,42) (Figure 1). Intuitively, this mechanism could only operate with the NTD of eIF3c stretching from the octamer base around the platform all the way to the 40S P-site (Figure 4B).

To complete the list of eIF3 interactions with other eIFs, mammalian eIF3a and eIF3c were, besides eIF1, also proposed to interact with eIF1A (24), and human eIF4G was (in contrast to yeast) shown to contain two distinct binding sites for eIF3, one of which contacts eIF3c and -d subunits, whereas the other binds eIF3e (75) (Figure 3A). These interactions were proposed to promote mRNA binding to the 40S ribosome in the eIF4G-dependent manner. eIF3a interacts with eIF4B (76) and its CTD, together with the eIF3b-RRM, associates with the initiation-specific non-processive mammalian helicase DHX29, and disruption of either contact impairs the DHX29 activity (77). It was proposed that DHX29 and eIF3 cooperate to promote scanning on structured mRNAs and to ensure stringency of AUG recognition (78), which is consistent with the eIF4B–eF3a contact and our previous yeast genetic data on g/TIF35 and a/TIF32 (38,39).

## THE eIF3 ROLE IN SELECTIVE mRNA TRANSLATION INITIATION

Besides the indispensable role of eIF3 in general translation initiation, there is a growing number of reports suggesting that eIF3 also controls alternative modes of translation initiation on cellular transcripts. The Cate's lab used the PAR-CLIP technique to identify transcripts that specifically interact with eIF3 in human 293T cells (79). They identified ∼500 mRNAs falling into distinct groups like cell cycle, apoptosis and differentiation, the 5′ UTRs of which specifically crosslinked to eIF3a, b, d and g subunits. Specific structural elements were predicted to feature in the 5′ UTRs of these mRNAs and drive eIF3-specific, cap-dependent activation or inhibition of translation initiation (79). This indicates that (a) eIF3 is capable to directly promote mRNA recruitment to 43S PICs, in accord with our earlier report from yeast cells (33), and (b) that it might do so in a highly selective, mRNA-specific manner and thus contribute to a wide variety of translational control mechanisms. Later the same group showed that one of these eIF3 subunits, namely eIF3d, under specific conditions even directly contacts the 5′ cap (80). Using the mRNA encoding the cell proliferation regulator c-JUN as the model mRNA representing the entire ‘crosslinked’ group it was proposed that the eIF3d cap-binding pocket interacts with the 5′ cap of the c-JUN mRNA only upon its allosteric activation by other eIF3 subunits and/or by the c-JUN 5′ UTR-specific secondary structure. Interestingly, this particular mRNA also contains the eIF4F-inhibitory element, which could prevent it from being initiated *via* the canonical 5′ cap/eIF4F-dependent pathway *in vivo*. Besides eIF3d, the non-essential octameric subunit eIF3l (25) was also shown to interact with the 5′ cap *in vitro*, but only in context of the entire mammalian eIF3 (81). Even though the cap-binding activity of eIF3d was not detected in the latter study (81), together these findings suggest an intriguing possibility that eIF3 interacts with numerous mRNAs encoding regulatory proteins in various modes involving the 5′ cap and/or higher-order secondary structures and single-handedly mediates their binding to the 43S PICs to control their expression in response to various stresses and cellular signals. Recent findings of our laboratory suggest that human cells might besides the eIF3 holocomplex also contain several minor but still operational subcomplexes lacking for example eIF3d or eIF3l–k or eIF3e–d–l–k or eIF3h–l–k subunits (25). Hence it is conceivable that it could be the existence of these subcomplexes that stands behind the eIF3 modularity in gene-specific mRNA recruitment to the PICs. In fact, the imbalance in the expression levels of individual eIF3 subunits, often seen in cancers and other pathologies (for review see (82,83)), might dysregulate this modular mRNA expression profile, as well as the ability of the palette of eIF3 subcomplexes to contribute to stress adaptation, and as such lie behind many of these medical conditions.

In support, the *Schizosaccharomyces pombe* cells lacking therein non-essential eIF3e and eIF3d subunits failed to synthesize components of the mitochondrial electron transport chain, leading to a defect in respiration, endogenous oxidative stress, and premature aging (84). The cells managed to maintain the energy balance by a switch to anaerobic glycolysis with increased glucose uptake and strict dependence on a fermentable carbon source. Since human eIF3e, which is essential in higher eukaryotes, was also suggested to promote translation of metabolic mRNAs, the authors proposed that eIF3—*via* its eIF3d-eIF3e module—might orchestrate an mRNA-specific translational mechanism controlling energy metabolism that could be disrupted in cancer. Other recent examples of the selective mRNA regulatory role of eIF3 are: (1) a report showing that loss-of-function mutations in the non-essential genes encoding eIF3k and eIF3l subunits result in a 40% extension in lifespan and enhanced resistance to endoplasmic reticulum (ER) stress in *Caenorhabditis elegans* (85); (2) two studies together demonstrating that eIF3 interacts with YTHDF1, an N(6)-methyladenosine reader protein that recognizes the m(6)A-modified mRNAs, to promote their translation in a cap-independent manner as an alternative mechanism to IRES-mediated initiation under various stresses when the cap-dependent pathway is suppressed (86,87); (3) a work suggesting that eIF3e promotes binding of the eIF4E-specific kinase Mnk1 (MAPK signal-integrating kinase 1) to eIF4G to induce eIF4E phosphorylation that might regulate selective mRNA translation; and finally (4) eIF3h was proposed to specifically contribute to modulating a lens development in zebrafish by regulating translation of lens-associated crystallin isoform mRNAs most likely *via* their UTRs (88).

## THE eIF3 ROLE IN IRES-MEDIATED INITIATION

Hepatitis C virus (HCV) and classical swine fever virus (CSFV) mRNAs contain related (HCV-like) internal ribosome entry sites (IRESs). Initiation on HCV-like IRESs relies on their specific interaction with the 40S subunit, which places the initiation codon into the P site, where it directly base-pairs with eIF2-bound Met-tRNA_i_^Met^ to form the 48S PIC. Importantly, only a subset of eIFs is needed for IRES-driven initiation; in fact different IRES classes have different requirements for eIFs (for review see (89)). It has been long known that eIF3 binds to the IIIabc four-way junction domain of the HCV IRES and is essential for its function in translation initiation (90–92). Earlier cryo-EM reconstructions suggested that the HCV IRES extends across eIF3 from the left arm to the right leg (60), which was later supported by demonstrating that the RNA-binding HLH motifs in eIF3a and eIF3c make direct contacts with the HCV IRES (93). Initially, it was believed that eIF3 actively promotes HCV IRES-mediated initiation (94), despite the fact that the ribosomal positions of eIF3 and the HCV IRES overlap. Our recent cryo-EM study of the CSFV-eIF3–40S complex resolved this paradox by showing that although the CSFV IRES interactions with the eIF3-bound 40S subunit were similar to those of the HCV IRES in the 40S-IRES binary complex, the eIF3 octamer was completely displaced from its typical ribosomal location and instead interacted with the apical region of the IRES domain III (95). Therefore, we proposed that the HCV-like IRESs actually prevent ribosomal association of eIF3 (at least that of the octamer because the YLC was not resolved) in their favor to be able to occupy the otherwise common 40S-binding site. As a consequence, they would also reduce formation of cellular PICs by sequestering eIF3 on viral PICs, thereby favoring translation of viral mRNAs. Hence, instead of being an IRES-translation-promoting factor, eIF3 may serve as an IRES-inhibitor. Actually, a negative role of eIF3 in a viral replicative cycle—but of a different kind—was also suggested by a study exploring the human immunodeficiency virus (HIV). It was shown that the eIF3d subunit inhibits the HIV replication and as such represents one of the targets-to-be-destroyed by the HIV protease, which specifically cleaves it during viral proliferation (96). On the other hand, binding of eIF3 together with PABP to the X-linked inhibitor of apoptosis (XIAP) IRES was recently shown to potentiate the ribosome recruitment to this IRES and thus to promote this IRES-driven translation (97). Given the diversity of the so far identified IRESs classes, it is conceivable that eIF3 may have stimulatory effects on some of them and inhibitory on some others—much like it was proposed for the structured cellular mRNAs (79). Precise biochemical analysis of each individual case are needed to corroborate especially those proposed molecular mechanisms that for their most part rely on structural and/or high-throughput studies only.

## THE eIF3 ROLE IN TRANSLATION TERMINATION, RIBOSOMAL RECYCLING AND STOP CODON READTHROUGH

The Pestova's lab revealed that after the ABCE1-mediated dissociation of post-termination complexes, the complete release of mRNA and deacylated P-site tRNA, which remain bound to post-termination 40S subunits, can be mediated by initiation factors eIF3, eIF1, eIF1A and the eIF3-associated factor eIF3j, at least *in vitro* (9,12). In detail, eIF1 with eIF1A relatively efficiently weakened the interaction of P-site deacylated tRNA with recycled 40S subunits, but complete dissociation of tRNA also required eIF3. In the absence of eIF3, eIF1/eIF1A-mediated release of P-site tRNA also led to mRNA dissociation; however, the presence of eIF3 resulted in less complete dissociation of mRNA from 40S subunits and efficient dissociation of mRNA required eIF3j. This is consistent with the stabilization role of eIF3 on mRNA binding (29,35), as well as with the reported negative cooperativity in 40S-binding between eIF3j and mRNA *in vitro* (28). Interestingly, at low concentrations of free Mg^2+^, eIF3, eIF1, eIF1A and eIF3j were sufficient to promote complete recycling of post-termination complexes even in the absence of ABCE1, in which case splitting of post-termination ribosomes was principally mediated by eIF3 (12).

These findings prompted us to test the role of eIF3 in ribosomal recycling and perhaps even in translation termination in living yeast cells. By measuring the frequency of stop codon readthrough in a collection of eIF3 mutants using an established dual-luciferase reporter assay, we found many mutations in all five yeast eIF3 subunits showing a significant reduction in readthrough (20). Conversely, deletion of the non-essential eIF3-associated factor *hcr1* (encoding eIF3j) resulted in significantly increased readthrough. Furthermore, we revealed that: (i) a substantial amount of eIF3, eIF3j and eRF3 specifically co-sedimented with terminating ribosomes isolated from RNase-treated heavy polysomes in an eRF1-dependent manner; (ii) eIF3 and eIF3j also occurred in ribosome- and RNA-free complexes with both eRFs and the recycling factor ABCE1/RLI1; (iii) the g/TIF35-NTD directly interacted with the N-terminal plus middle domain (N-M) of eRF1 and (iv) eIF3 mutations reducing readthrough genetically interacted with mutant eRFs in a manner indicating that eRFs and eIF3 have antagonistic role at the same stage of the termination pathway. Hence, we proposed that wild-type eIF3 binds terminating ribosomes, perhaps in a complex with both eRFs, where it modulates the precision of stop codon recognition by eRF1 in order to fine tune the termination process (Figure 1).

In addition, we observed that the *hcr1* deletion resulted in accumulation of eRF3 in heavy polysomes in a manner suppressible by overexpressed ABCE1/RLI1, to which it directly binds to (98), and that high dosage of ABCE1/RLI1 fully suppressed the slow growth phenotype of *hcr1Δ*, as well as its termination but not initiation defects (20). Hence, we suggested that upon stop codon recognition, yeast eIF3j facilitates eRF3•GDP ejection from the post-termination complexes to allow binding of its interacting partner ABCE1/RLI1 near the A-site-based eRF1 to proceed with polypeptide release and ribosomal recycling. The fact that eIF3j was shown to reside next to eIF1A sitting near the 40S A-site (65), in agreement with earlier biochemical and genetic evidence suggesting that it spans across the mRNA entry channel (28,37), is consistent with this model. Importantly, these data implied that the termination function of eIF3j is more critical for optimal proliferation than its function in translation initiation, at least in yeast.

Interestingly, the cooperation of eIF3 and eIF3j with ABCE1 may easily reach beyond the termination phase. The recent structural studies revealed that ABCE1 interacts with the intersubunit face of the 40S subunit at the universally conserved GTPase binding site even after ribosomal recycling (71,72), and most likely participates in the initiation phase hand-in-hand with eIF3 and other eIFs, as proposed earlier (99). We proposed that ABCE1 could act as an anti-association factor preventing subunit joining until the AUG codon has been recognized by Met-tRNA_i_^Met^ and eIFs occurring at the ribosomal interface have been cleared away to allow formation of the 80S initiation complex (72).

The unexpected eIF3 role in termination sparked our curiosity to characterize its molecular basis; surprisingly, we revealed that it critically promotes programmed stop codon readthrough (Figure 1) (21). It *de novo* associates with pre-termination complexes, where it apparently interferes with the eRF1 decoding of the third/wobble position of any of the three stop codons set in the unfavorable termination context, thus allowing incorporation of near-cognate tRNAs with a mismatch at the same position. It is important to note that the eIF3 role in programmed readthrough was found to be conserved between yeast and humans (21). The precise molecular mechanism is still unknown; however, at least two possibilities come to mind. First possibility is based on the fact that a portion of the eIF3 body projects into the vicinity of the mRNA entry channel and several of its subunits interact with RNA (2,45,46,59,64,68); eIF3 could directly interact with the readthrough-promoting sequences surrounding the stop codon and perhaps even mediate their effects on shifting the equilibrium of stop codon recognition by eRF1 *versus* near-cognate tRNAs on the side of the latter.

**Figure 5.**
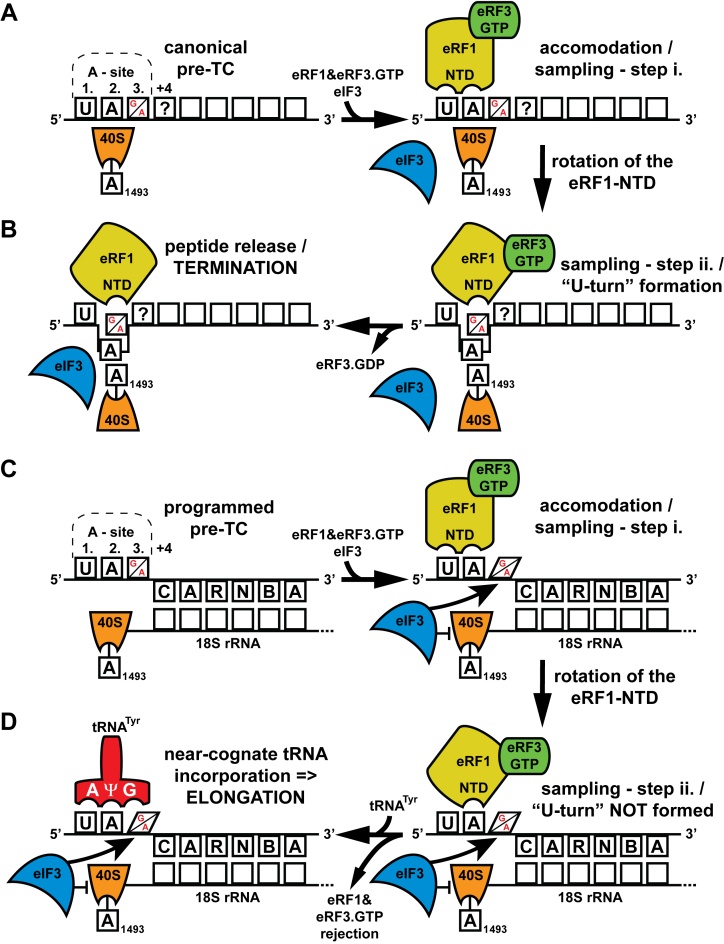
Translation initiation factor eIF3 promotes programmed stop codon readthrough - revised model adapted from (21). (**A** and **B**) Canonical termination; stop codon in the termination favorable context appears in the A-site (only UAG and UAA stop codons are indicated for illustration purposes; UGA works by the same mechanism; +4 base is indicated by a question mark), eRF1 in complex with eRF3.GTP binds to it and samples the codon in a two-step process (100). In the first step, the first and second nucleotides of the stop codon are recognized by specific residues of the eRF1-NTD. This is followed by the eRF1-NTD conformational re-arrangement during the second step, which probably includes flipping the A1493 base (according to the *E. coli* nomenclature) accompanied by formation of the U-turn-like conformation (103,104). This step permits decoding of the third nucleotide. As a result, eRF1 stably accommodates in the A-site triggering GTP hydrolysis on eRF3, followed by polypeptide release and ribosomal recycling. eIF3 has minimal, if any role here (see text for further details). (**C** and **D**) Programmed stop codon readthrough; stop codon occurs in the unfavorable termination context bearing specific consensus sequences like CAR-NBA in its 3′ UTR—in this particular case proposed to base-pair with 18S rRNA (160). The eIF3 presence in the pre-termination complex—perhaps in co-operation with these sequences—prevents the A1493 phosphate group to flip and thus specifically interferes with the proper decoding of the third position of programmed stop codons (19,21). This results in ejection of the eRF1–eRF3•GTP complex from the pre-termination complexes allowing incorporation of near-cognate tRNAs with the mismatch at the third position to read through the stop codon and continue with elongation. For details please see the main text.

The second possibility emanates from the proposal that the canonical stop codon recognition by eRF1 occurs in two steps (100). It is known that when the eRF1–eRF3•GTP complex enters the A-site with the stop codon in it, the pre-termination complex undergoes major conformational re-arrangements particularly at the A-site and around the mRNA entry channel (101–104). Part of these re-arrangements concerns the eRF1-NTD, which flips the 18S rRNA nucleotide A1493 (*Escherichia coli* nomenclature) so that it stacks on the second and third stop codon bases (103,104) (Figure 5A and B, right-handed panels). As a result, stop codon adopts the eukaryotic-specific U-turn-like conformation within a decoding pocket formed by the eRF1-NTD and the ribosome that is now also capable of accommodating the +4 base; i.e. the base immediately following the stop known to have a significant impact on the efficiency of readthrough (see for example (15,19)). To combine this U-turn-like stop codon tetranucleotide idea with the older, and in our opinion still valid, two-step model, we propose the following. In the first step, the first and second nucleotides of the stop codon are recognized by specific residues of the eRF1-NTD (Figure 5A). This is followed by the eRF1-NTD re-arrangement during the second step, which might actually include flipping the A1493 base accompanied by formation of the U-turn-like conformation, permitting decoding of the third nucleotide (Figure 5B). As a result, eRF1 stably accommodates in the A-site triggering GTP hydrolysis on eRF3. Where does eIF3 stand in this model?

Intriguingly, the A1493 nucleotide, which is required for efficient decoding by eRF1, is also targeted by the aminoglycoside paromomycin. This heavily-studied readthrough inducing drug, when bound to the termination complexes, displaces the A1493 phosphate group within the stop codon decoding pocket and deforms the near-cognate codon–anticodon helix in the A-site (105). As a result, the terminating ribosome does not actively sense the correct Watson–Crick base-pairing geometry and thus does not discriminate against near-cognate tRNAs; in other words relaxes the termination fidelity. Since the stimulatory effect of paromomycin on programmed stop codon readthrough is epistatic with that of eIF3 (19,21), it is tempting to speculate that eIF3 could also interfere with A1493. It could for example prevent its flipping, which most probably occurs during the transition from the first to the second step (Figure 5A and B, right-handed panels), and thus specifically interfere with the proper decoding of the third position of programmed stop codons (Figure 5C and D, right-handed panels), which was observed (single mismatches at the first or second stop codon positions showed no genetic interactions with eIF3) (19,21). This would lead to the ejection of the eRF1–eRF3•GTP complex from the pre-termination complexes allowing near-cognate tRNAs with a mismatch at the same position to incorporate into the A-site and continue elongating (Figure 5D, left-handed panel). How could eIF3 influence the position of A1493? Either directly, since it was proposed to reach the A-site *via* the a/TIF32-CTD (50,66,70) and interact with the N-M domain of eRF1 *via* its g/TIF35 subunit (20), or allosterically by binding to constituents of the decoding pocket such as RPS2/uS5 and RPS3/uS3 (38,39).

In fact, an earlier study carried out with the eRF1–eRF3•GMPPNP complex bound to terminating ribosomes may provide a solid support for the latter option. It was shown that another important part of the termination complex re-arrangements involves a movement of helix 16 (h16) of 18S rRNA and the NTD of RPS3/uS3 toward each other, which results in the establishment of a new head–body connection on the solvent side of the 40S subunit and a constriction of the mRNA entrance (106). Binding of the no-go mRNA decay complex DOM34–HBS1 to stalled yeast ribosomes also led to the appearance of a density bridging h16 and RPS3/uS3 (107). Strikingly, conformational changes involving 18S rRNA helices 16, 18 and 34, as well as RPS3/uS3 occur also during translation initiation and are controlled by a cooperative action of the eIF1, 1A, 2(TC) and 3 (66,108). First, binding of these eIFs dissolves the mRNA entry channel ‘latch’ formed by h18 in the body of the 40S and h34 and RPS3/uS3 in the head to open the channel for mRNA loading and subsequent ribosomal scanning. The reversal latch closure on AUG recognition, which is again triggered by the delicate interplay between eIF1, 1A, 2, 3 and 5 and involves the same ribosomal components (66,108), then clamps on the mRNA and arrests scanning (reviewed in (1)). This ‘initiation’ latch closure markedly resembles that provoked by eRF1–eRF3•GMPPNP during termination (106). Since mutations in the eIF3a-CTD (binds RPS3/uS3 and h16–18) and eIF3g (binds RPS3/uS3) confer phenotypes indicating destabilization of the closed PIC conformation (as the means of reducing start codon recognition), as well as phenotypes suggesting the opposite effect of destabilizing the open conformation of the PIC (as they appeared to reduce the processivity of scanning), it was proposed that the eIF3a-CTD and eIF3g regulate the transition between scanning-conducive and scanning arrested conformations (38,39). Therefore, it is very tempting to consider that in case of termination, the constriction at the mRNA entrance tunnel may include similar actors and serve the similar purpose; i.e. to clamp onto the mRNA to stabilize the termination complex as it prepares for peptide release. If true, multiple contacts that eIF3a and eIF3g establish with the constituents of the decoding pocket during this process could allosterically impact the position of the A1493 phosphate group in the decoding pocket, which would antagonize the conformational changes required for proper stop codon recognition by eRF1 and allow readthrough.

## THE eIF3 PROSPECTIVE ROLE IN NONSENSE-MEDIATED DECAY (NMD) PATHWAY

Besides canonical stop codons, readthrough can also occur on premature stop codons (PTCs). There it is closely connected with the Nonsense-mediated decay (NMD) pathway, as majority of mRNAs containing PTCs are destined to degradation (109). Considering the eIF3 involvement in readthrough, it is no surprise that eIF3 has also been implicated in NMD (Figure 1) (110). According to one model, aberrant termination at a PTC occurring upstream of a post-splicing exon junction complex (EJC) in mammals results in UPF1-bridging the contact between eRFs and the EJC-associated UPF2/UPF3, which is followed by the SMG1-mediated phosphorylation of UPF1 triggering a series of downstream events. Based on *in vitro* experiments with human reconstituted system it was proposed that phospho-UPF1 set in the PTC termination complex directly interacts with eIF3 supposedly bound to the 48S PIC on the same mRNA molecule, and that this ‘looping’ interaction prevents formation of the elongation-competent 80S complex; i.e. initiation on this aberrant mRNA is repressed (110). Noteworthy, a recent proteomics study confirmed that eIF3 interacts with UPF1; however, independently of its phosphorylation status (111). Nonetheless, taken into account the eIF3 roles in termination events it remains to be unambiguously demonstrated that UPF1 really interacts with initiating and not terminating eIF3 and blocks initiation on NMD mRNA substrates by this proposed mechanism in living cells. Purely theoretically, the eIF3’s critical role in readthrough and reinitiation (see below) could speak for the opposite effect of eIF3 on NMD; i.e. for its inhibition.

A growing number of studies suggest that translational repression is one of the key steps that precedes mRNA delivery to the degradation machinery (112), which would support the first option; i.e. the UPF1-signalled, eIF3-mediated initiation arrest as an attractive mechanism by which eIF3 contributes to efficient degradation of PTC-containing mRNAs by NMD. In support, the eIF3g (113) and eIF3e (114) knockdowns were shown to strongly inhibit NMD, and immunoprecipitation experiments showed that eIF3e co-purifies with UPF2 and the ‘pioneer round’ 5′ cap-binding protein CBP80. In addition, it was shown that several transcripts known to be upregulated by UPF1 or UPF2 depletion were also found to be stabilized when eIF3e was suppressed (114).

Major support for the second, NMD-inhibition option comes from the other well established model of NMD called the faux 3′ UTR-mediated NMD. Here the NMD is triggered by aberrant translation termination at stop codons located in an environment of the mRNP that is devoid of signals necessary for proper termination—like the presence of poly(A)-binding protein (PABP), which interacts with both eRFs and thus prevents their binding to UPF1 (115). Using tethering assays it was demonstrated that the three RRMs of human PABPC1, which mediate the PABC1 interaction with the eIF4G-NTD, were sufficient to antagonize NMD. Since tethering of the eIF4G-NTD, as well as of the eIF4G core in direct contact with eIF3 also suppressed NMD, the authors proposed that PABPC1, eIF4G and eIF3 directly cooperate with both eRFs in translation termination and NMD suppression (116). In fact, co-operation between eIF3, and PABPC1 and eIF4G (looping both mRNAs ends together) was suggested to lie behind the NMD-resistance conferred by mRNAs containing short uORFs (so-called the ‘AUG-proximity effect’). First it was demonstrated that eIF3f and h subunits are required to prevent NMD of β-globin reporter transcripts with AUG proximal PTCs (117). Next the Romao's group proposed that simultaneous binding of PABPC1 to the poly(A) tail, as well as to the eIF4G/eIF3-bound early elongating ribosome, brings PABPC1 to close proximity with the termination complex allowing it to interact with eRF3, which in turn impairs the UPF1-eRF3 interaction and thus inhibits NMD (118). There is still much to be learnt about the eIF3 role (positive or negative) in NMD. It is entirely possible that some eIF3 subunits stimulate whereas some other inhibit NMD and the final outcome depends on the type and position of individual PTCs, which may employ different means of NMD-driven regulatory mechanisms (like EJC-mediated *versus* faux 3′ UTR-mediated NMD) under different conditions.

To conclude this and the previous chapters, we propose that in case of PTCs in the readthrough unfavorable context occurring in the false termination neighborhood and/or upstream of EJCs, eIF3 could block new rounds of initiation on defective mRNAs by a UPF1-mediated mechanism described by (110). However, if these PTCs are set in the readthrough favorable context, the eIF3 binding to pre-termination complexes could override the NMD-triggering signals and promote efficient readthrough to allow synthesis of a full-length protein by a molecular mechanism that normally operates on genuine, programmed stops and is outlined above. In case of genuine but non-programmed stop codons and short uORFs, eIF3 can prevent NMD in co-operation with other ‘canonical termination-signaling’ factors to stabilize the mRNA.

## THE eIF3 PRESENCE ON EARLY ELONGATING RIBOSOMES AND ITS ROLE IN REINITIATION

Translation reinitiation (REI) is a gene-specific regulatory mechanism that takes place on the same mRNA molecule after translation of an upstream ORF (usually very short) followed by incomplete ribosomal recycling; i.e. only the large 60S subunit and deacylated tRNA are recycled (Figure 1) (reviewed in (2,7,14)). The efficiency of canonical REI depends on: (i) *cis*–acting mRNA features surrounding a given short uORF; (ii) duration of the uORF elongation; (iii) some eIFs involved in the first initiation event and (iv) the intercistronic distance needed for the acquisition of the new TC (reviewed in (2)). It has been well established that uORFs are relatively widespread across all eukaryotic genomes (13%, 30%, 44% and 49% of yeast, *A. thaliana*, mouse, and human transcripts, respectively, contain uORFs (119–121)), and that most of them inhibit expression of the main ORF (some of them very severely) by completing the ribosomal recycling step after their translation. There are, however, exceptions in the so-called REI-permissive uORFs that inhibit the expression of the main ORF only very modestly, if at all. These are rarely alone, in fact they often precede REI-non-permissive uORFs or uORFs overlapping with the main ORF, and as such create mRNA-specific regulatory mechanisms modifying the expression of the main ORF in response to various environmental stimuli. Expression of various growth factors, transcription factors and other proto-oncogenes, proteins involved in differentiation, development, cell cycle, stress response, learning and memory can be found to be regulated *via* REI. Hence, it is no surprise that uORF polymorphism has also been implicated in a variety of human diseases (119,122,123).

The first hint that eIF3 may directly promote translation reinitiation came from the cauliflower mosaic virus (CaMV) polycistronic RNA (124). The cauliflower mosaic virus transactivator, TAV, was shown to physically interact with eIF3 (*via* its eIF3g subunit) and the 60S subunit to mediate efficient recruitment of eIF3 to polysomes, allowing translation of polycistronic mRNAs by reinitiation; however, after translation of long ORFs in this case. The eIF3g subunit stimulates REI also in the budding yeast *S cerevisiae* (38). Later the *Arabidopsis thaliana* eIF3h subunit was demonstrated to be required for REI efficiency of specific 5′ mRNA leader sequences containing series of upstream open reading frames like in case of the transcription factor ATB2/AtbZip11; eIF3h supposedly ensures that a fraction of uORF-translating ribosomes retain their competence to resume scanning for downstream REI (125,126). In support, the kinase cascade of the plant ortholog of mammalian target-of-rapamycin (mTOR) and S6 kinase (S6K) phosphorylates and thus activates eIF3h, which then contributes to efficient loading of uORF-containing mRNAs onto polysomes and their expression *via* the REI mechanism (127).

Besides eIF3g, REI in yeast is critically promoted also by eIF3a (45). Genetic experiments carried out with the mRNA leader of the yeast transcriptional activator GCN4, which combines two REI-permissive uORFs with two non-permissive uORFs in an intricate fail-safe mechanism (128), revealed the following with respect to role of yeast eIF3 in REI. (Note that the *GCN4* regulatory mechanism responding to stress-induced changes in the TC levels is reviewed elsewhere (128–130).)

Both highly REI-permissive uORFs of the *GCN4* mRNA (uORF1 and uORF2) contain so-called REI-promoting elements (RPEs) upstream of their AUGs, some of which fold into specific structural features like hairpins and stem loops (46,128). In detail, uORF1 utilizes four RPEs (i–iv), whereas uORF2 separately utilizes only a single RPE v (similar in sequence with the uORF1-specific RPE i) and, in addition, ‘shares’ RPE ii with uORF1. We first showed that the RPEs i and iv of uORF1 and RPE v of uORF2 genetically interact with two separate segments encompassing amino acid residues 51–60 (nicknamed Box6) and 161–170 (Box17) of the a/TIF32-NTD (45,46). Mutating the latter RPEs or these eIF3a-Boxes severely reduced REI permissiveness of both uORFs and combining these mutations revealed genetic epistasis (46), suggesting that these molecules closely co-operate in promoting efficient REI. Taken into account a favorable location of the eIF3a-NTD on the 40S subunit next to the mRNA exit channel (50,65–67), we proposed that the eIF3a-Boxes 6/17 directly contact these RPEs that have, upon termination on uORF1 or uORF2, already emerged from the exit pore and became solvent-exposed (46). This interaction would be instrumental in preventing full ribosomal recycling by stabilizing the mRNA•40S post-termination complex to enable its subsequent resumption of scanning and reinitiation downstream (Figure 1). This would mean, however, that eIF3 has to either stay bound to 80S ribosomes elongating these short uORFs or leave the ribosome upon subunit joining but immediately come back as a part of the termination/recycling complex.

The former idea that some REI-specific eIFs remain transiently associated with elongating ribosomes and that increasing the uORF length or the ribosome transit time increases the likelihood that these factors are dropped off was not new (7,131). Besides our yeast genetics, *in vitro* experiments with mammalian reconstituted systems also suggested that eIF4F (particularly the eIF4G’s central one-third fragment interacting with eIF3 and eIF4A), and presumably eIF3 as well, persistently interact with the post-initiation ribosomes for a few elongation cycles to stimulate resumption of scanning of the post-termination 40S subunit (132). It was shown that if the splitting of post-termination complexes proceeded in the presence of eIFs 3, 1, 1A and eIF2-TC *in vitro*, 40S subunits remained on mRNA and reinitiated at nearby upstream or downstream AUGs; imposing the 3′-directionality additionally required eIF4F (133). The eIF3–eIF4G co-operation in this process is easily conceivable because these two factors interact with each other (75,134) and both of them have a favorable location on the solvent-exposed side of the small subunit (2,59,65,66,135). However, direct *in vivo* evidence for their involvement in the establishment of the REI competence has been lacking and the molecular details of their REI-promoting role have been unclear.

To address this critical issue, firstly from the eIF3 point of view, and clearly distinguish between the aforementioned two possibilities (i.e. does eIF3 stay bound to elongating 80S ribosomes or does it come off before elongation commences and back on upon termination?), we recently developed a new *in vivo* RNA–protein Ni^2+^ pull down (Rap-Nip) assay. This yeast assay captures 80S ribosomes bound by initiation factors, in our case by eIF3, while translating and terminating on short uORFs (47). Using this *in vivo* assay we demonstrated that eIF3 does travel with early elongating ribosomes at all *GCN4* uORFs regardless their permissiveness for REI and gradually falls off as the length of any of these uORF grows. In support, recent ribosomal profiling experiments revealed the so-called ‘5′ ramp’ of ribosomes at the beginning of the coding regions that was attributed to the engagement of some eIFs, particularly eIF3, with the 80S ribosome during early elongation transiently slowing down its rate (analogous to early transcription elongation) (136).

**Figure 6.**
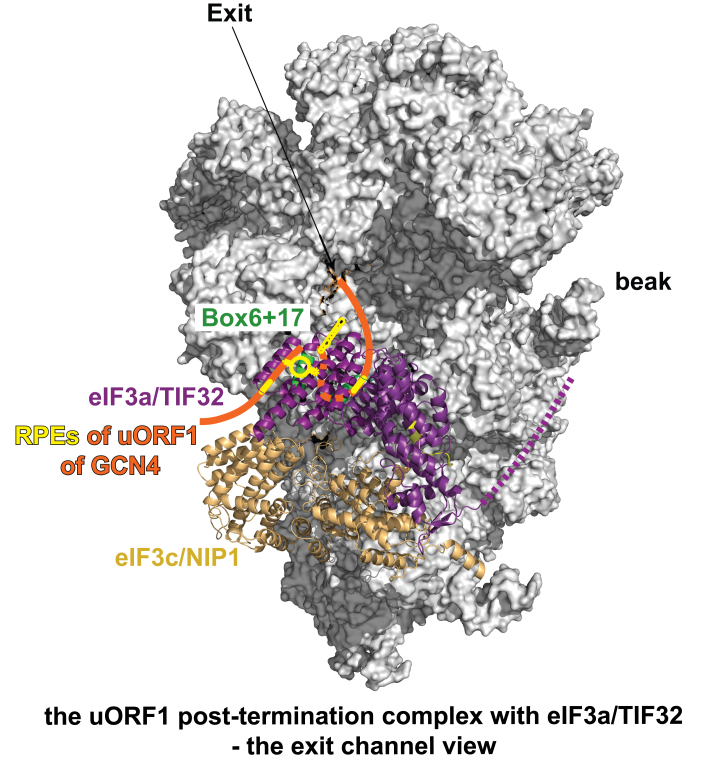
Graphical illustration (adapted from (47)) of the proposed arrangement of the post-termination complex on uORF1 with its RPEs interacting with Box 6 and Box 17 segments of the N-terminal domain of a/TIF32 to promote resumption of scanning for REI on *GCN4*. The exit channel view of the py48S-closed complex shows only two incomplete eIF3 subunits for simplicity: c/NIP1 in wheat and a/TIF32 in purple with its extreme NTD in light purple and its C-terminal HCR1-like domain (HLD) represented by a dotted line (its structure is unknown and thus its placement in the py48S complex was only predicted). The location of both a/TIF32 boxes is marked in green; the 5′-UTR of uORF1 is shown in orange with its RPEs depicted in yellow-orange.

In case of *GCN4*, we propose the following model. During scanning for and translation of the REI-permissive uORFs, RPEs progressively fold into specific secondary structures that, upon termination, eIF3 interacts with *via* eIF3a-Box6/17 to stabilize the 40S subunit on the uORF1 (or uORF2) stop and prevent it from full recycling (Figure 6) (46,47). Thanks to that, the post-termination 40S subunit can, upon acquisition of other essential eIFs, resume scanning for REI downstream (Figure 1). Whether eIF4G also contributes to this process is currently under investigation. How yeast g/TIF35 participates in this mechanism is also unknown, except that its RRM domain, which occurs near the mRNA entry and not exit channel, does not stimulate REI in cooperation with any of known *GCN4 cis*-acting features (38).

To examine whether there is any mechanistic resemblance in the REI *modus operandi* between yeast and mammals, we have very recently analyzed the flanking sequences of the REI-permissive uORF1 from the mRNA leader of the human *GCN4* functional homolog, *ATF4*, encoding stress-inducible transcriptional activator, the regulation of which is governed in a similar fashion to that of *GCN4* (137). We revealed that its 5′ sequences contain two well conserved structural features resembling the *GCN4‘*s RPEs. We also showed that the basic level of REI that the *ATF4*’s uORF1 allows is significantly increased by an independent contribution of both uORF1 flanking sequences, and that human eIF3h, like in plants, seems to stimulate efficient REI on *ATF4*. Whether it also interacts with the uORF1 5′ sequences like eIF3a does in yeast, however, remains to be determined (138).

eIF3 was also implicated in the mechanism of an exceptional case of termination/reinitiation after translation of a long ORF that is best described for the polycistronic mRNA of feline calicivirus (139,140). A specific 87-nt element (called TURBS for termination upstream ribosome binding site) preceding the overlapping termination/initiation site of two long ORFs folds into a special secondary structure that base-pairs with a specific segment of 18S rRNA and, at the same time, interacts with eIF3 *via* its several subunits including eIF3a and eIF3g. This intricate network of interactions should prevent dissociation of the mRNA/eIF3/40S complex in order to allow efficient REI on ORF3. However, whether or not the *in vivo* role of eIF3 in this process is critical must be further verified, because recent *in vitro* experiments showed that the post-termination ribosomal tethering of mRNA by TURBS diminishes dependence on the eIF3-mediated reinitiation by the post-termination 40S subunits, and instead allows reinitiation by the post-termination 80S ribosomes (141).

## eIF3 IN HUMAN HEALTH AND DISEASE

Deregulation of eIF3 expression and/or function has been proposed to play either a causal role or at least contribute to the etiology of various diseases including cancer, neurodegenerative states etc.; some eIF3 subunits were even suggested to serve as oncogenes or tumor suppressors with potential prognostic values. The purpose of this chapter is not to cover all aspects of the prospective eIF3 involvement in various human diseases but to briefly discuss some of the discoveries reporting reduced or increased expression of various eIF3 subunits in the context of what we have recently learned about the human eIF3 integrity and potential consequences of imbalanced expression of its individual subunits (25). In other words, it has been long known that various types of cancer and other diseases are associated with altered expression levels of most, if not all, eIF3 subunits (for review see (82,83)). However, we recently demonstrated that these perturbations to the relatively balanced expression of eIF3 subunits lead to the formation of partial eIF3 subcomplexes that are associated with defects in the rate of translation and cell fitness. This may suggest that the observed pathological effects often attributed to alterations in the expression levels of a single eIF3 subunit are actually caused not only by the lack or excessive amount of this particular subunit *per se*, but by the loss or gain of function of partial eIF3 subcomplexes that could form in cancer and other sick cells as a result of these expression anomalies.

Perhaps the easiest interpretations of the functional consequences of altered expression levels in disease can be made with the eIF3d subunit, the siRNA-mediated knock down of which impact neither the expression levels of other eIF3 subunits nor the integrity of eIF3 *in vivo*, nonetheless confers severe defects in growth and translation rates (25). eIF3d was shown to be overexpressed in muscle invasive disease and ovarian cancer, whereas the eIF3d knock-down in metastatic T24M bladder cancer cells inhibited cell proliferation, migration, and colony formation *in vitro* and decreased tumor growth in xenograft models (142,143). eIF3d was also shown to be associated with hepatocellular carcinoma where it was up-regulated as a direct consequence of Hepatitis delta virus replication (144). Given what was said above, it is likely that these phenotypes are directly associated either with (i) malfunctioning of free eIF3d protein that occurs in excess to the rest of eIF3, or (ii) malfunctioning of eIF3 lacking its d subunit in these carcinoma cells.

The eIF3l subunit was found to interact with the Flavivirus NS5 and eIF3l overexpression was suggested to promote Flavivirus translation and thus to modulate the yellow fever virus replication cycle (145). Depletion of endogenous eIF3k de-sensitized simple epithelial cells to various types of apoptotic stimuli and promoted the retention of active caspase 3 in cytoplasmic inclusions by increasing Cas3 binding to keratins (146). Since knock downs of eIF3k and eIF3l mutually impact only their own expression and are dispensable for the integrity of the rest of the eIF3 complex (25), the above described phenotypes should be attributed to the changed expression levels of not only one of these subunits but to both of them. In accord, it was recently shown that the loss-of-function mutations in the *Caenorhabditis elegans* genes encoding eIF3k and eIF3l resulted in an identical phenotype; i.e. in a 40% extension in lifespan and enhanced resistance to endoplasmic reticulum (ER) stress (85).

Similarly, knock down of eIF3h leads to a concurrent downregulation of only the eIF3k and l subunits, hence observations that eIF3h is highly amplified for example in breast and prostate cancers (147) may imply that changes in the expression levels of eIF3k and l might be also expected, which could cause undesirable changes in expression profiles of numerous mRNAs contributing to malignancy.

eIF3e was perhaps the first eIF3 subunit the altered expression of which was connected with cancer (148). In particular, the introns of its gene were found to be a frequent integration site of mouse mammary tumor virus (MMTV) provoking changes in the eIF3e expression in an ‘intron-specific’ manner. For example, the MMTV integration at intron 6 resulted in a decreased expression of eIF3e in several human breast and lung carcinomas (149). We recently learnt that when eIF3e is significantly underexpressed, the expression of eIF3d, eIF3k and eIF3l is also dramatically reduced (25). As a result, the eIF3 subcomplex lacking only these four subunits forms, which might be responsible for the progression of the observed cancer phenotypes. On the other hand, the MMTV integration at intron 5 produced a truncated eIF3e protein with a malignant transformation potential that could, if still able to incorporate into eIF3, easily result from altered functional properties of the entire eIF3 complex (150).

The siRNA down regulation of either eIF3c or eIF3f or eIF3m subunits pretty much destroys the entire octamer preserving only the YLC (eIF3a–b–g–i) module (25), which is still capable to promote very basic eIF3 functions that are, however, insufficient to support life of human cells (41). Hence any reports describing pathological phenotypes stemming from altered expression of these three subunits should be at least partially regarded as a failure of the dramatically compromised eIF3 complex to ensure productive general translation initiation in sick cells; like underexpression of eIF3f in gastric, melanoma and pancreatic cancers supposedly deregulating apoptosis (151,152), eIF3f ubiquitination and proteosomal degradation during muscle atrophy (153), overexpression of eIF3m in human cancer cell lines and colon cancer patient tissues (154), as well as overexpression of eIF3c in testicular seminoma cells (155).

There are numerous reports implicating altered expression of eIF3a and eIF3b in numerous types of cancer (for review see (82,83)). However, knock down experiments with both of these subunits in HeLa and HEK293T cells clearly revealed that their expression is absolutely essential for formation and stability of the entire eIF3. As aforementioned, they serve as the nucleation core without which eIF3 does not assemble, and changes in their expression have dramatic impact on expression of all other eIF3 subunits (25). Hence, from this point of view we think that all these reports should be considered with caution and interpret the observed pathological anomalies as a consequence of the radically crippled eIF3 complex.

## CONCLUDING REMARKS

Whereas the subunit composition of both forms of eIF3 (5-subunit Sc-eIF3 vs. 12-subunit m-eIF3) is now clear, individual initiation roles, as well as the assembly pathways have been elucidated, there is much to be learnt from the structural point of view. Several snapshots of eIF3 with or without other eIFs bound to PICs have been taken and provided an extremely valuable insight into particular initiation steps. However, we still do not know how both eIF3 forms look like free in solution, what structural rearrangements they have to undergo when they associate with other eIFs and mainly during their initial contact with the small ribosomal subunit.

We have learnt the mechanics of the mRNA entry channel latch opening and closure (1). However, we still do not understand when and how the eIF3a-CTD–eIF3b–i–g module swings to the ribosomal interface side, what it does there with respect to mRNA loading, scanning and AUG recognition (in particular considering its interactions with eIF2γ and eIF1 in the decoding center), and when it eventually comes back. Similarly, the mechanics of the eIF3c-NTD involvement in AUG recognition together with the P-site-based eIF1 and eIF5, the precise ribosomal placement of which remains a mystery, needs to be explored in the near future. Besides the need to reveal the eIF5 localization within the PICs, there is a similarly pressing need to identify a precise position of the eIF4F factors, as well as of PABP. The still improving methods of RNA interference and antisense approaches to knock down specific proteins, newly established CRISPR–Cas9 technology, revolutionized Cryo-electron microscopy, as well as the burst of specialized ribosomal profiling studies that are now available to capture scanning 48S PICs (156) should also help by a great deal in addressing most of these questions.

Apart from the initiation phase, it will be extremely valuable to determine how eIF3 associates with terminating ribosomes and uncover molecular details of its role in fine-tuning the termination fidelity and in promoting stop codon readthrough or reinitiation. What factors eIF3 really interacts with during these events and what are the molecular consequences of these contacts? For example, does eIF3 interact with eIF4F to promote reinitiation? What is the functional interplay between ABCE1 and eIF3 during ribosomal recycling followed by a new round of initiation? How does eIF3 manipulate the decoding pocket during termination to promote incorporation of near-cognate tRNAs to the A-site? What is the molecular mechanism of the eIF3 involvement in NMD? Does it vary with the placement and nature of the stop codon across the entire length of mRNA, under stress *versus* normal condition, etc.?

Considering the growing evidence of the eIF3 involvement in the transcript-specific translation regulation, it will also be important to identify all mRNAs that are subject to this eIF3-specific regulation, as well as other contributing factors (mRNA features and proteins). eIF3 was some time ago implicated in signal transduction pathways by recruiting protein kinases such as mTORC1 and S6K to the surface of the 40S subunit (157,158), which we did not cover here. It is entirely possible that signal transduction pathways may govern or at least markedly impact this peculiar role of eIF3.

And last but not least, taking into account numerous reports implicating eIF3 in cancer incidence, metastasis development, prognosis and therapeutic response, we should foster our effort to clarify the exact mechanism of the eIF3 involvement in oncogenesis and enlighten its real chances in cancer treatments, as these areas of research are indeed of the supreme interest for human health.
